# Zfp36l1 establishes the high‐affinity CD8 T‐cell response by directly linking TCR affinity to cytokine sensing

**DOI:** 10.1002/eji.202350700

**Published:** 2023-12-07

**Authors:** Georg Petkau, Twm J. Mitchell, Marian Jones Evans, Louise Matheson, Fiamma Salerno, Martin Turner

**Affiliations:** ^1^ The Babraham Institute Babraham Research Campus Cambridge United Kingdom

**Keywords:** CD8 T cells, RNA binding proteins, T cell activation, TCR affinity

## Abstract

How individual T cells compete for and respond to IL‐2 at the molecular level, and, as a consequence, how this shapes population dynamics and the selection of high‐affinity clones is still poorly understood. Here we describe how the RNA binding protein ZFP36L1, acts as a sensor of TCR affinity to promote clonal expansion of high‐affinity CD8 T cells. As part of an incoherent feed‐forward loop, ZFP36L1 has a nonredundant role in suppressing multiple negative regulators of cytokine signaling and mediating a selection mechanism based on competition for IL‐2. We suggest that ZFP36L1 acts as a sensor of antigen affinity and establishes the dominance of high‐affinity T cells by installing a hierarchical response to IL‐2.

## Graphical abstract

In summary, we identify the RNA binding protein ZFP36L1 to act as a sensor of antigen affinity and to establish the dominance of high‐affinity T‐cell clones by promoting the response to cytokines like IL2.

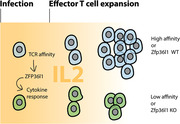

## Introduction

Upon antigen encounter T cells interpret multiple cues including costimulatory molecules and pro‐ and anti‐inflammatory cytokines and translate these into molecular programs which lead to clonal expansion and differentiation [1]. A diverse repertoire of T cells with varying affinities for antigen, and thus different requirements for activation, compete amongst each other for antigen, costimulation, cytokines, and nutrients [2, 3]. The outcome of this competition is that few high‐affinity T‐cell clones dominate at the peak of the T‐cell response.

Multiple mechanisms have been proposed to control the expansion and differentiation of an antigen‐specific CD8 T‐cell population [4, 5]. These include the frequency of homotypic cell contacts determined by cell density and the consequent impact of inhibitory receptors expressed by the expanding population [4, 5]. In addition, intercellular communication via local gradients of cytokines such as IL‐2 has been suggested to be essential to adjust the clonal sizes of effector and memory populations [4, 6]. Access to IL‐2 can be licensed by other inflammatory cytokines including type‐I IFNs and IL‐12 which enhance clonal expansion and effector differentiation [7, 8]. It has also been shown that strongly activated T‐cell clones are able to enhance the proliferation of weakly activated clones by the production of an excess of IL‐2, at least in vitro [9]. Using the expression of Nur77‐GFP as a reporter for TCR signaling, a threshold for activation has been suggested to induce cell proliferation and to be invariable to different, above‐threshold, stimulation strengths [10]. Costimulatory cues, including IL‐2, can lower cumulative activation thresholds of T cells whereby these signals are integrated by transcription factors that drive metabolic reprogramming, proliferation, and differentiation [11, 12, 13]. Interestingly, the timing of initiation of cell division, acquisition of effector function, and the underlying transcriptional pathways were not dependent on CD8 T‐cell affinity [14]. In line with this, stimulation of naïve OT‐I cells with lower affinity antigens in vivo did not result in differential initial expansion of T cells early in the response [15]. High‐affinity T cells establish their larger clone size rather by additional rounds of cell division [15]. In the later phase of the immune response T‐cell proliferation is driven by IL‐2 and the prolonged expression of CD25, driven by inflammatory cytokines [8, 16]. In addition, inflammation has been suggested to rescue high‐affinity but not low‐affinity T cells from apoptosis when antigen is limited [17]. Currently, we lack a mechanistic understanding of how T‐cell affinity is translated into the differential response to cytokines and preferential expansion of high‐affinity clones.

Autocrine and paracrine IL‐2 are to some extent redundant as autocrine IL‐2 is largely dispensable for primary CD8 T cell expansion in vivo [18, 19, 20], but its production by other cells is essential [16, 19, 21–23]. Access to IL‐2 is critical to regulate apoptosis in a TCR‐dependent fashion resulting in the preferential survival of high‐affinity clones [24]. Thus competition for IL‐2 by different T‐cell clones and subsets may be essential to shape the T‐cell immune response and the establishment of dominance by high‐affinity T‐cell clones [2, 25–27]. It is to date not clear how T cells of different affinities which are recruited into the immune response and thus have overcome thresholds of activation establish the duration of the response to IL‐2. How TCR affinity is directly linked to the sensitivity to IL2 during an immune response remains unknown.

The ZFP36 family of RNA binding proteins is best characterized for their role as suppressors of cytokine production. Three paralogues (*Zfp36*, *Zfp36l1*, and *Zfp36l2*) are expressed by T cells where they have been shown to limit cytokine production, and more recently, to regulate metabolic and transcriptional programs [28, 29, 30, 31]. CD8 T cells lacking both *Zfp36* and *Zfp36l1* displayed enhanced in vitro expansion, accelerated differentiation, and more potent effector function. Moreover, ZFP36 and ZFP36L1 enforced dependence on CD28 costimulation. ZFP36L2 has a unique role in repressing cytokine production by memory T cells [28], but because of an apparent redundancy between the *Zfp36* paralogues [29, 30, 31] unique roles for individual ZFP36 family members in T‐cell activation and differentiation have yet to be identified. In the present study, we show that ZFP36L1 acts as a node that senses TCR‐ligand affinity and promotes sensitivity to cytokine signals, thereby organizing interclonal competition and safeguarding the selection of high‐affinity T‐cell clones.

## Results

### ZFP36 and ZFP36L1 confer a competitive advantage to CD8 T cells in vitro

**Figure 1:**
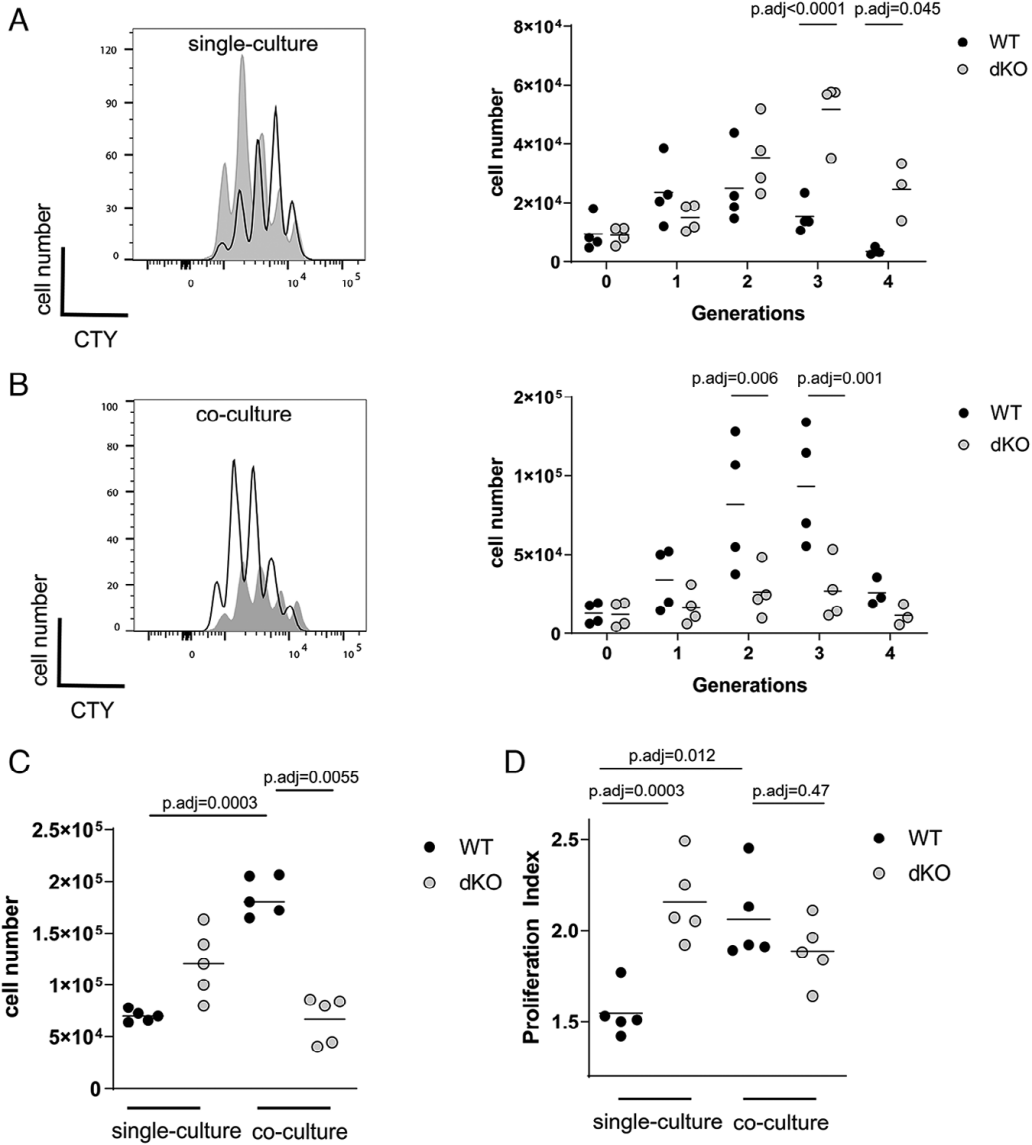
ZFP36 and ZFP36L1 confer a competitive advantage to CD8 T cells in vitro. (A, B) Left panels show the dilution of CTY by naïve WT (open) and dKO (filled) OT‐I cells stimulated with N4 peptide for 48 h individually (A) or in co‐culture (B). Right panels show the absolute cell numbers per generation in single (A) and co‐cultures (B): Data is compiled from four independent experiments. Each data point is representative of one biological replicate. (C) Absolute cell numbers of WT and dKO OT‐I cells after 48 h with N4 peptide in individual and co‐culture. Data is compiled from five independent experiments. (D) Proliferation index of cells as in panel (C). Statistical significance in (A, B) was determined by two‐way ANOVA followed by Bonferroni multiple comparisons test. Statistical significance in (C, D) was determined by one‐way ANOVA followed by Tukey's multiple comparisons test. Cell numbers in co‐cultures are adjusted to the double culture volume to compare to WT culture conditions, maintaining the same cell culture density. The statistical mean is indicated by horizontal line in (A, B).

Naïve CD8 T cells from OT‐I TCR transgenic (TCR specific for the SIINFEKL peptide from chicken ovalbumin) CD4^cre^ ZFP36^fl/fl^ ZFP36L1^fl/fl^ mice, lacking *Zfp36* and *Zfp36l1* in T cells (from now on termed dKO) proliferate more extensively than OT‐I cell isolated from CD4^wt^ ZFP36^fl/fl^ ZFP36L1^fl/fl^ littermate controls, after in vitro stimulation with high‐affinity SIINFEKL (N4) peptide. When cultured alone they accumulate substantially greater numbers of cells that had undergone more cell divisions after 48 h (Fig. 1A). Since both RNA‐binding proteins (RBPs), ZFP36 and ZFP36L1, have been previously shown to limit autocrine cytokine production including IL‐2 [30, 31], which could feedback as costimulatory signals we wanted to evaluate whether the greater expansion is due to increased production of cytokines by the dKO. Therefore, we performed co‐culture experiments where both cell types are exposed to the same environment and can interact with each other. In co‐cultures after 48 h of stimulation, expanding WT cells showed a similar distribution of cells per generation as the dKO but accumulated more cells per generation (Fig. 1B). Thus, when exposed to the same environment, dKO cells lost their superior ability to expand.

WT cells co‐cultured with dKO cells accumulated greater cell numbers than the dKO cells and these numbers exceeded those of WT cells cultured alone (Fig. 1C). Similar results were obtained with nontransgenic naïve CD8 T cells stimulated with anti‐CD3 and anti‐CD28 (Fig. S1A, B). The proliferation index, a measure of cell‐intrinsic capacity to divide, of WT cells is increased when co‐cultured with dKO cells and reaches similar levels as single cultured dKO cells (Fig. 1D). The cell division of the dKO cells is only very slightly reduced upon co‐culture, suggesting the reduced numbers of dKO cells in the presence of WT cells reflects increased cell death. Thus, when WT and dKO cells were cultured in the same well, the dKO cells were less able to compete with their WT counterparts.

### RBP promotes T‐cell fitness by enhancing the response to IL‐2

**Figure 2:**
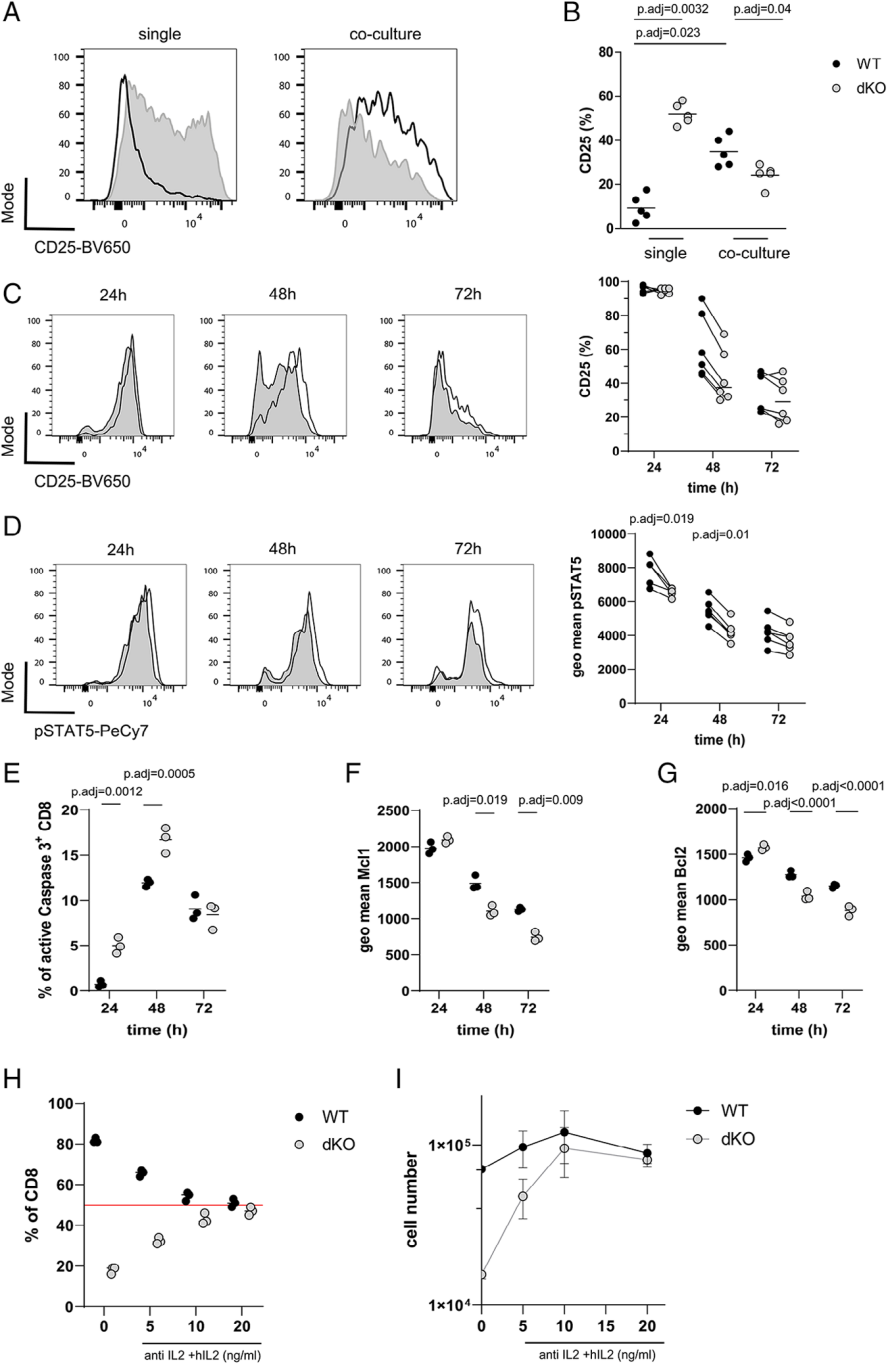
RBP promote T‐cell fitness by enhancing the response to IL2. (A) Representative histograms showing CD25 surface expression in WT (open) and dKO (filled) OT‐I cells in single and co‐cultures, 48 h after N4 peptide stimulation. (B) Frequency of CD25^+^ WT and dKO OT‐I cells in single and co‐cultures. Each data point represents one biological replicate. Data is compiled from five independent experiments. (C) Left panel shows a time course analysis of intracellular CD25 expression as representative histograms of cocultured WT and dKO OT‐I cells, which were stimulated with N4 peptide for indicated times. Right panel shows frequency of CD25‐positive cells. (D) Left panel shows representative histograms of pSTAT5 over indicated times after N4 peptide stimulation of WT and dKO co‐cultured OT‐I cells. Right panel shows the geo mean of pSTAT5 expression per cell. Statistical significance was determined by two‐way ANOVA followed by the Bonferroni test for multiple comparisons. Data is compiled from two out of three independent experiments with each data point representing a technical replicate. (E) Frequencies of active‐Caspase3 positive OT‐I cells in co‐cultures stimulated with N4 peptide. Geo mean fluorescence intensity of (F) Mcl1 and (G) Bcl2 in co‐cultured WT and dKO OT‐I cells is shown. Data is representative of two in (E) and three in (F, G) independent experiments with each data point representing a technical replicate. Statistical significance was determined by one‐way ANOVA followed by Tukey's test for multiple comparisons. Frequencies (H) and absolute cell numbers (I) of WT and dKO co‐cultured OT‐I cells, 72 h after stimulation with N4 peptide in normal medium or in the presence of anti‐mouse IL‐2 antibody and indicated amounts of added human IL‐2 from the beginning of the culture. Data is representative of two independent experiments with each data point representing a technical replicate. The statistical mean is indicated by horizontal line in all panels. Error bars indicate the SD of the mean in all panels.

To assess the response to IL‐2, which is critical for T‐cell survival and proliferation in vitro, we activated WT and dKO OT‐I cells with high‐affinity N4 peptide for 48 h alone or in co‐culture. When cultured alone WT cells expressed low levels of CD25, the high affinity subunit of the IL‐2 receptor, when compared with dKO cells. Upon co‐culture, CD25 was increased on WT cells, but its expression was reduced on dKO cells compared with single cultures (Fig. 2A, B). The greater expression of CD25, when dKO cells are cultured alone, likely reflects the increased amount of IL‐2 produced since the expression of CD25 can be induced by IL‐2 [32]. When in co‐culture and exposed to similar amounts of IL‐2, CD25 expression increases (Fig. 2B) and remains higher in WT cells compared with dKO, which suggests decreased IL‐2 signaling in dKO cells.

Next, we assessed the dynamics of IL‐2 responsiveness following antigen activation. To assess the total CD25 protein (present on the surface and intracellularly), we stained for intracellular CD25 and phosphorylated pSTAT5 (pY694) as a marker of IL‐2 signal transduction in co‐culture. Twenty‐four hours after activation, the frequencies of CD25^+^ cells were the same between WT and dKO, and pSTAT5 was only slightly reduced in dKO (Fig. 2C, D). At 48 h after activation, both the frequency of CD25^+^ cells and the phosphorylation of STAT5 were reduced in the dKO compared with WT OT‐I (Fig. 2C, D). There was a higher frequency of active Caspase3‐positive dKO cells than WT cells 24 and 48 h after activation (Fig. 2E). This was accompanied by a reduced expression of the antiapoptotic proteins MCL1 and BCL2 in dKO cells compared with WT cells, at later timepoints following activation (Fig. 2F, G, Fig. S1C, D). The increased apoptosis in dividing dKO cells may reflect diminished STAT5 activity and lesser amounts of antiapoptotic proteins.

The reduced expression of CD25 and decreased signaling via STAT5 in dKO cells in coculture prompted us to suspect that dKO cells are inferior in processing IL‐2 signals when in competition with WT cells. We therefore controlled the concentration of IL‐2 present in the cultures using anti‐mouse IL‐2 blocking antibody (JES6‐1A12) while providing different concentrations of human IL‐2 (hIL‐2) which is not bound by JES6‐1A12. Adding increasing amounts of hIL‐2 normalized the ratios of WT and dKO, reaching 50% when hIL2 was added at 20 ng/ml (Fig. 2H). The absolute cell numbers recovered from these cultures 72 h after activation were also comparable between WT and dKO at high doses of hIL‐2 (Fig. 2I). This data shows that when amounts of IL‐2 are low the survival of the dKO cells is compromised due to reduced IL‐2 responsiveness.

### Competitive fitness for cytokines is controlled by RBPs during bacterial infection in vivo

**Figure 3:**
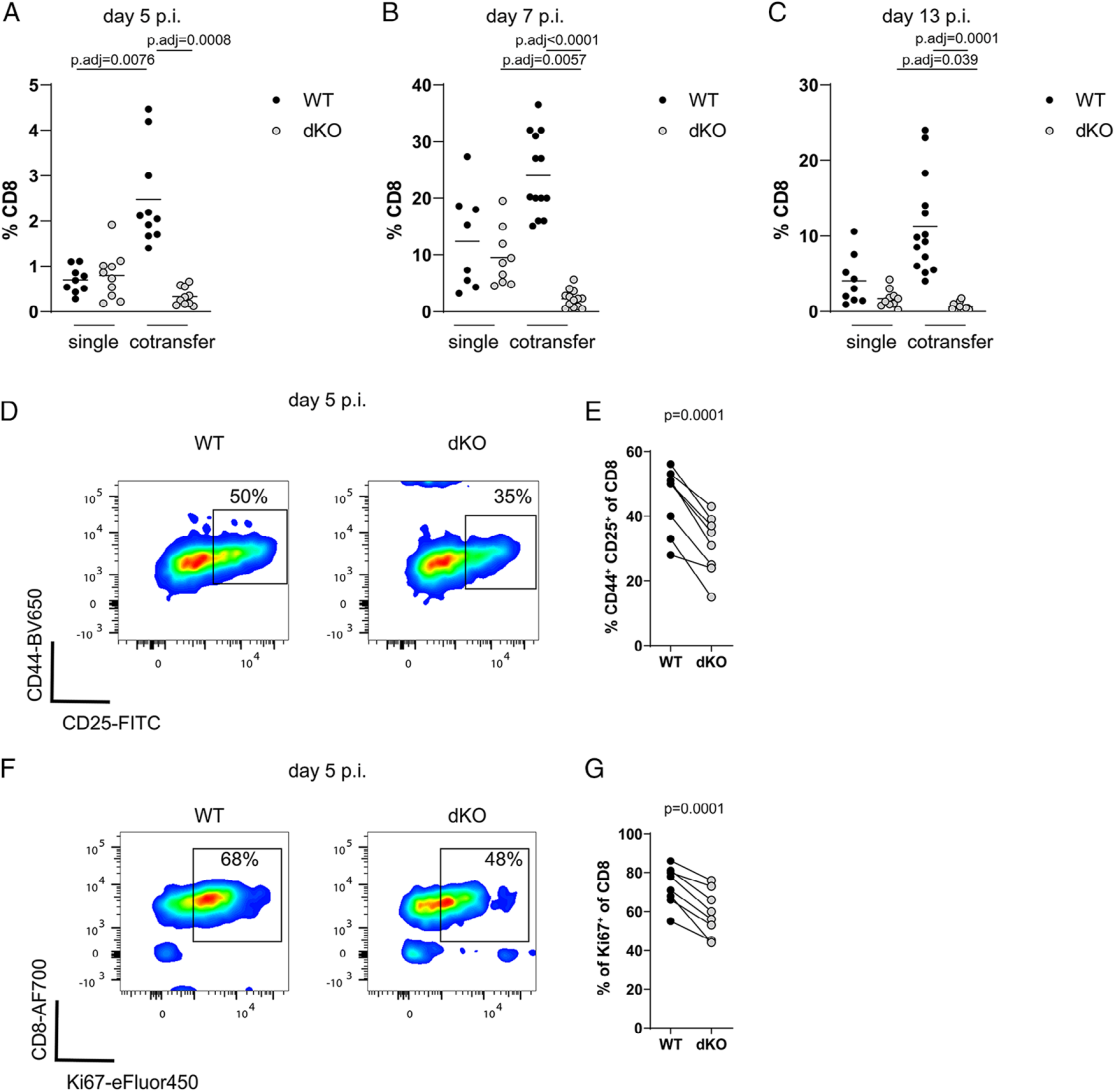
Competitive fitness for cytokines is controlled by RBPs during bacterial infection in vivo. (A) Frequencies of WT and dKO OT‐I cells single or co‐transferred in the blood of recipients on day 5, 7 (B), and 13 (C) postinfection with *attLm*‐OVA. Data was pooled from three independent experiments. Statistical significance was determined by two‐way ANOVA followed by Tukey`s test for multiple comparisons. (D) Representative FACS plots showing expression of CD44 and CD25 on co‐transferred WT and dKO OT‐I cells in spleens of recipient mice on day 5 post‐*attLm*‐OVA infection. (E) Frequency of CD44^+^CD25^+^ OT‐I T cells. (F) Representative FACS plots showing expression of CD8 and Ki‐67 on co‐transferred WT and dKO OT‐I cells. (G) Frequency of Ki67^+^ OT‐I T cells. Statistical significance in panel (E) and (G) was determined using a paired *t*‐test. The statistical mean is indicated by horizontal line in (A–C).

To examine the fitness of dKO cells in a competitive setting in vivo, we transferred 500 WT (CD45.1^+^) and dKO (CD45.2^+^) naive OT‐I cells into recipients in which the host cells were positive for both CD45.1 and CD45.2. In parallel, to generate controls with the same number of high‐affinity antigen‐specific cells, we transferred 1000 WT or dKO naïve OT‐I cells into separate hosts. We followed the expansion of donor OT‐I cells in the blood after infection with an attenuated strain of ΔAct *Listeria monocytogenes* expressing chicken Ovalbumin (OVA_257–264_ (N4)) (*attLm*‐OVA). During the expansion phase of the primary response individually transferred WT and dKO OT‐I cells showed comparable frequencies in blood in contrast to the in vitro proliferation results. When co‐transferred, the frequencies of WT OT‐I cells were increased by the presence of dKO cells (Fig. 3A–C). Moreover, the proportion of dKO OT‐I cells in blood was reduced compared with co‐transferred WT OT‐I cells indicating the dKO cells expand less when in competition (Fig. 3A–C). Furthermore, the numbers of dKO OT‐I cells were diminished when co‐transferred with WT OT‐I compared with single dKO OT‐I cell transfers at the peak of the response and during contraction (Fig. 3A–C). The diminished ability of dKO cells to compete was strikingly apparent at day 13 when dKO cells are 20‐fold less frequent than their WT counterparts.

Following co‐transfer of WT and dKO OT‐I cells and subsequent infection with *attLm*‐OVA, the frequencies of WT OT‐I cells expressing CD25 on day 5 postinfection, were higher than that of dKO cells in the spleens of recipient mice (Fig. 3D, E). Furthermore, the frequency of Ki‐67^+^ cells, a marker of proliferating cells, was reduced amongst dKO cells compared with WT cells (Fig. 3F, G). Expression of CD25 and IL‐2 signaling extend the time of proliferation of CD8 T cells during the later phase of the primary response [8, 16, 22]. In line with this, our data suggests that the dKO cells exit the cell cycle earlier in vivo. Maintenance of CD25 expression and proliferation at this stage has been attributed to the action of type I IFNs and IL‐12 [8]. The addition of IL‐12 to WT and dKO OT‐I cells cultured in vitro increased CD25 expression and only partially restored the ability of dKO cells to expand in competition (Fig. S2A, B). Other cytokines produced by T cells including IFNγ have been shown to be important regulators of expansion and differentiation during the primary response to *L. monocytogenes* infection [33]. Therefore, we tested the potential contribution of TNF and IFNγ to the expansion defects of dKO cells in vitro. We blocked IFNγ or TNF in cocultures of WT and dKO cells stimulated with peptides and did not observe an effect on the reduced ability of dKO cells to expand in the presence of WT cells (Fig. S2C). Thus, these inflammatory cues are insufficient to compensate the cell‐intrinsic expansion deficit of T cells lacking RBPs.

### ZFP36 and L1 suppress a network of cytokine signaling pathway regulators

**Figure 4:**
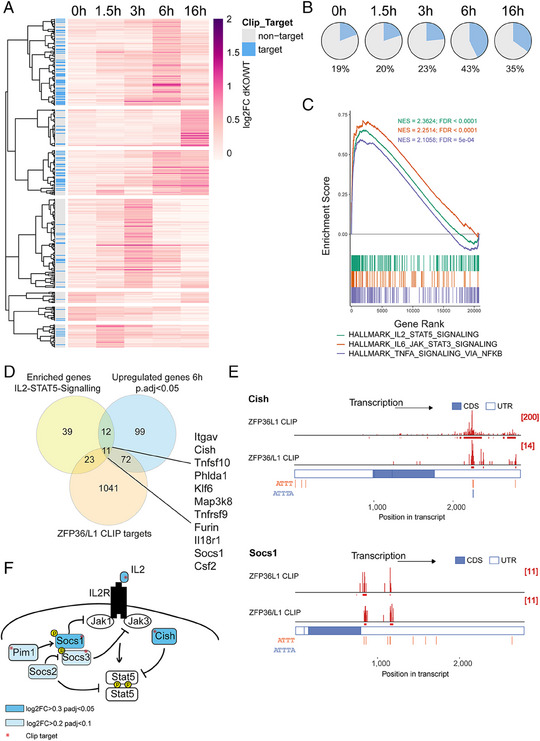
ZFP36 and ZFP36L1 suppress a network of cytokine signaling pathway regulators. (A) Heatmap using correlation as a measure of hierarchical distances, visualizes differentially upregulated genes (log2FC > 0.3, adjusted *p*‐value < 0.05) between naïve WT and dKO cells at indicated timepoints after stimulation with N4 peptide. Direct CLIP target transcripts are indicated in the left column. (B) Pie charts visualize the proportion of RBP targets among significantly differentially upregulated genes in dKO. (C) Gene set enrichment analysis, using the murine hallmarks gene collection, shows the enrichment score versus the gene rank of the top three positively enriched gene sets. A false discovery rate (FDR) is indicated for each gene set. (D) Venn diagram shows the shared genes that are contained in the positively enriched IL‐2‐STAT5 signaling hallmark gene set, the significantly upregulated genes at 6 h postactivation, and a list of RBP CLIP targets. (E) CLIP data showing the number and position of sequencing reads (in red) across *Cish* and *Socs1* transcripts (a set of top two lanes). In each set the top lane shows ZFP36L1 CLIP data from OT‐I CD8 CTLs stimulated for 3 h with N4 peptide; the bottom lane shows pan ZFP36 family CLIP data from in vitro‐activated naive CD4 T cells. ATTT and ATTTA which are the main DNA sequences encoding AU‐rich RNA motifs bound by ZFP36 and ZFP36L1 are identified in orange and blue. (F) Schematic view of RBP target genes involved in IL‐2 signaling. Asterisks indicate direct RBP targets. Genes with icons highlighted in blue are differentially increased in dKO versus WT during the stimulation time course.

To understand the mechanism by which ZFP36 and ZFP36L1 maintain responsiveness to IL‐2, we performed Next Generation RNA Sequencing of naïve WT and dKO OT‐I cells activated for 0, 1.5, 3, 6, and 16 h in vitro with N4 peptide. Differential gene expression analysis revealed several genes that were significantly increased in dKO OT‐I cells (Fig. 4A). Amongst the differentially increased transcripts, we identified those that were directly bound by the RBPs by examining a list of direct targets identified by RNA crosslink‐immunoprecipitation (CLIP) in activated CD4 (panZFP36) [34] and CD8 (ZFP36L1) [30] T cells (Table S1).

Most direct target genes were identified among genes that were differentially increased at 6 h postactivation, comprising more than 40% of the increased genes at this time point (Fig. 4A, B). Gene set enrichment analysis of Hallmark gene sets revealed IL‐2‐STAT5, IL‐6‐JAK‐STAT3, and TNF‐NF‐kB signaling pathways to be most significantly enriched among increased genes at this time (Fig. 4C). Within the positively enriched genes among the IL‐2‐STAT5 gene set, which were also targets of the RBP, was a subset of genes involved in regulating the cytokine response (Fig. 4D), including the family of suppressors of cytokine signaling, *Socs1* and *Cish* [35] (Fig. 4E). *Socs3* was also identified as direct CLIP target, although the increase in its expression did not reach significance (6 h timepoint, log2FC = 0.39, *p*.adj = 0.06) (Fig. S3A). *Socs1* is known to be induced by multiple cytokines and mediates negative feedback to limit their signaling [36]. SOCS‐1 [37] and SOCS‐3 [38] have been shown to be induced by IL‐2 and to inhibit IL‐2 signaling by interfering with JAK‐1 and JAK‐3 signal transduction. *Socs2*, which we have found to be slightly increased 6 h postactivation (log2FC = 0.23, *p*.adj = 0.02) but which is not a direct RBP target, has been suggested to promote IL‐2 signaling via degradation of SOCS‐3 [39] but also to suppress STAT5 phosphorylation in CD4 T cells [40]. The role of CISH with regard to its ability to inhibit STAT5 phosphorylation remains controversial and it may play a more prominent role in inhibiting TCR signaling [41, 42, 43]. We also identified *Pim1*, encoding a kinase that negatively regulates STAT5 signaling by stabilizing SOCS‐1 and SOCS‐3 [44], to be a direct RBP target (Fig. S3B) and to be slightly increased early in dKO OT‐I cells (3 h timepoint, log2FC = 0.32, *p*.adj = 0.06). In summary, we find a network of genes to be targeted by RBPs that suppress cytokine signaling (summarized in Fig. 4F).

### Ablation of *Socs1* restores competitive fitness of RBP deficient T cells

**Figure 5:**
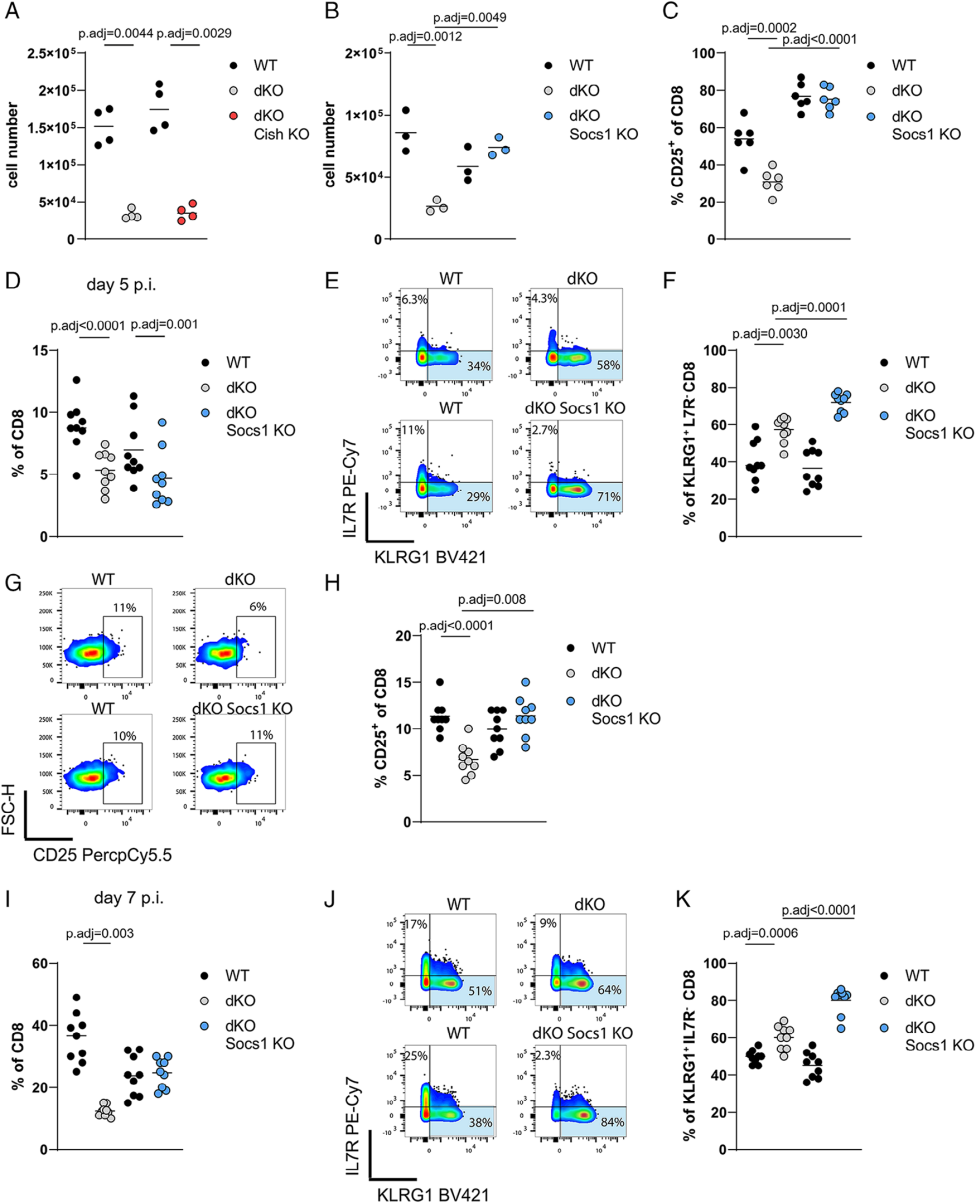
Ablation of *Socs1* restores competitive fitness of RBP deficient T cells. (A) Cell numbers of WT OT‐I co‐cultured with dKO OT‐I cells treated with nontargeting guide RNAs or dKO OT‐I cells where *Cish* or *Socs1* gene (B) has been deleted 72 h after stimulation with N4 peptide. Each dot in the panels represents a technical replicate. Representative data from three independent experiments is shown. (C) Frequencies of CD25‐positive CD8 T cells are shown. Each dot in the panel represents a technical replicate and data is pooled from two of three independent experiments. (D) Frequencies of total CD8 cells from co‐transferred WT OT‐I with either dKO OT‐I treated with nontargeting guide RNAs or dKO OT‐I cells where *Socs1* has been deleted in blood of recipient mice on day 5 after infection with *attLm*‐OVA. (E) Representative FACS plots and frequencies (F) of KLRG1^+^ cells in blood of mice 5 days after infection. (G) Representative FACS plots and frequencies (H) of CD25 positive cells. (I) Frequencies of total CD8 cells from co‐transfers in spleens of recipient mice on day 7 after infection with *attLm*‐OVA. j) Representative FACS plots and frequencies (K) of KLRG1^+^ cells in spleens/blood of recipient mice 7 days after infection. Statistical significance was determined by one‐way ANOVA followed by Tukey's test for multiple comparisons. The statistical mean is indicated by horizontal line in all panels.

The SOCS family proteins not only regulate cytokine responses, but some of its members such as *Cish* are also regulators of TCR signaling [42, 45]. We deleted *Cish*, using Cas9 RNPs in naïve dKO OT‐I cells (Fig. S3C) and tested whether the absence of regulation of TCR signaling mediated by CISH would restore the dKO T cell's competitiveness. However, co‐culture of WT OT‐I cells with *Cish* deficient dKO OT‐I cells did not rescue the dKO cells (Fig. 5A). Next, we tested whether releasing the suppression of cytokine signaling in dKO cells by deletion of *Socs1* would improve the fitness of dKO cells. We validated the deletion of *Socs1* by Cas9 RNPs using CTLs expanded in the presence of IL‐2 which revealed a reduction, but not a complete loss, of SOCS1 protein (Fig. S3D). Subsequent activation in co‐culture with WT OT‐I cells showed that dKO cells that received RNPs targeting *Socs1* expanded as well as WT OT‐I cells in co‐culture (Fig. 5B). Expression of surface CD25 on dKO cells deficient for *Socs1*, as well as on co‐cultured WT cells, was significantly increased (Fig. 5c). Thus, deletion of *Socs1* in RBP‐deficient T cells improves and prolongs their response to IL‐2 in co‐culture. This specific de‐repression of γ‐cytokine signaling is sufficient to render the dKO cells competitive.

Next, we tested whether the release of cytokine signaling blockade by the deletion of *Socs1* in RBP deficient T cells restores their ability to compete with WT T cells in vivo. After co‐transfer of WT and dKO OT‐I cells treated with nontargeting guides or WT and dKO OT‐I cells in which *Socs1* was deleted, we infected the recipient mice with *attLm*‐OVA and followed the expansion of OT‐I cells on day 5 in blood and day 7 in the spleen. On day 5, we found no clear effect of *Socs1* deletion on the expansion of dKO cells, which were still inferior to their WT counterparts (Fig. 5D). Cells that were lacking *Socs1* had accumulated greater frequencies of KLRG1^+^ short‐lived effector cells (SLEC; Fig. 5E, F). Moreover, these cells showed increased expression of CD25 which is consistent with prolonged IL‐2 signaling in these cells (Fig. 5G, H). The formation of SLEC is enhanced by the IL‐2‐CD25 signaling axis [16, 46], suggesting that in the absence of *Socs1*, the greater formation of SLEC is secondary to enhanced cytokine signaling. By day seven postinfection, at the peak of the response, dKO OT‐I cells lacking *Socs1* had expanded to the same extent as their WT counterparts, thus they no longer displayed a competitive disadvantage (Fig. 5I). Also, on day 7, cells lacking *Socs1* had greater frequencies of KLRG1^+^ SLEC (Fig. 5J, K). Overall the deletion of the RBP target *Socs1* in RBP deficient cells restores their competitive fitness allowing these cells to compete for IL‐2 and potentially other cytokines such as IL‐12 [47] and IL‐15 [48] in vivo. In addition, the deletion of *Socs1* shows an additive effect on acceleration of SLEC formation by dKO cells. This exacerbation may result from the action of cytokine‐sensitive transcription factors which we previously identified as RBP targets that drive effector cell differentiation [30].

### *ZFP36L1* plays a nonredundant role in mediating high‐affinity T‐cell expansion

To investigate how ZFP36 and ZFP36L1 contribute to T‐cell competition, we first assessed their expression in response to stimulation with peptides of varying affinity for the OT‐I TCR. To trace the expression of both RBPs at single‐cell resolution, we generated reporter mice in which open reading frames encoding the fluorescent proteins mAmetrine or mCherry were inserted in frame with the start codon of *Zfp36* and *Zfp36l1* respectively (Fig. S4A, B). In these reporter mice, *mAmetrineZfp36* and *mCherryZfp36l1* are under the same transcriptional control as endogenous *Zfp36* and *Zfp36l1*. We validated that expression of ^mAmetrine^ZFP36 and ^mCherry^ZFP36L1 followed the same transient expression kinetics as the endogenous proteins using in vitro expanded and restimulated CD8 cells from OT‐I ^mAmetrine/+^ *Zfp36* ^mCherry/+^ *Zfp36l1* heterozygous mice (Fig. S4C, D).

**Figure 6:**
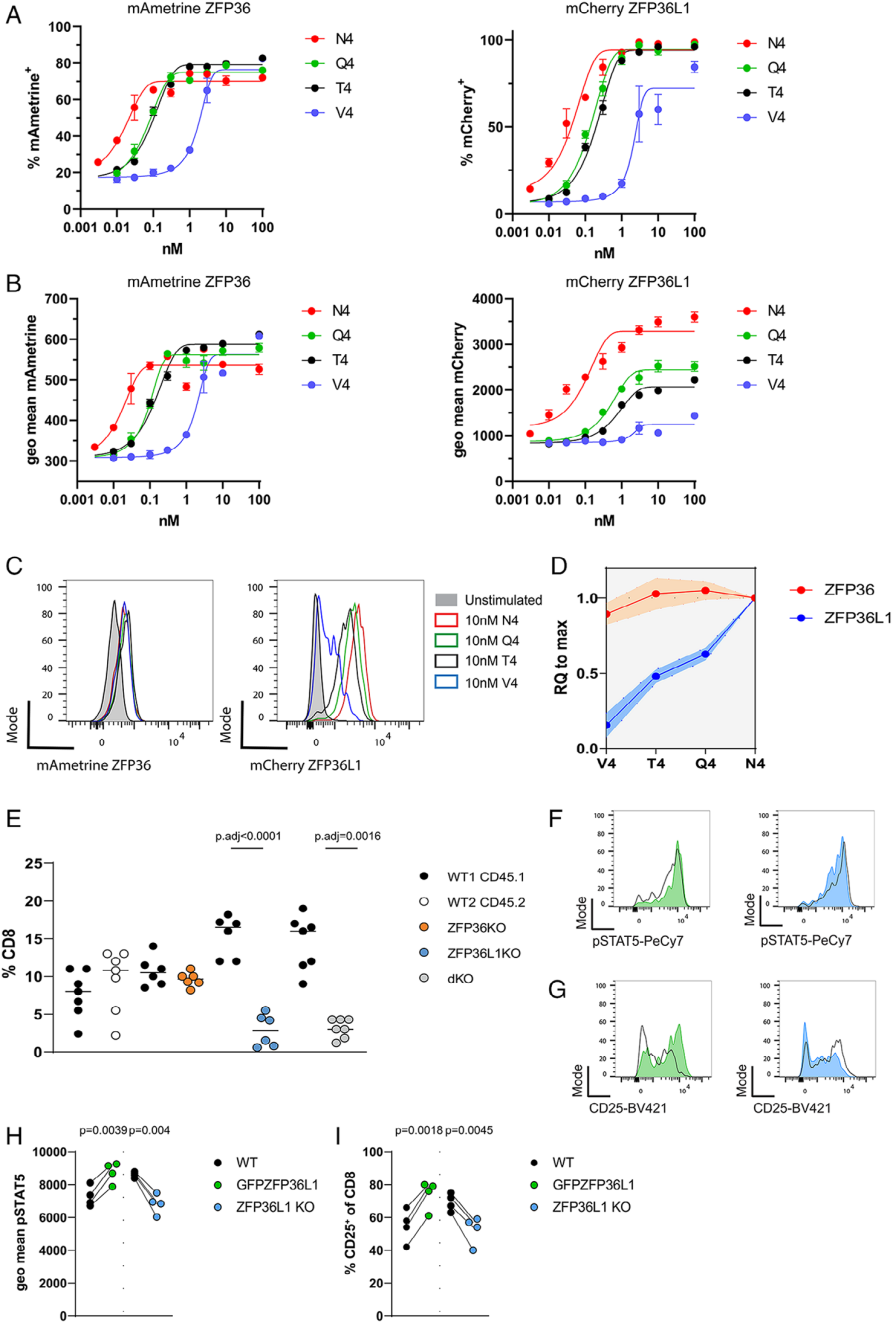
ZFP36L1 plays a nonredundant role in mediating high‐affinity T‐cell expansion. (A) Frequencies of ^mAmetrine^ZFP36^+^ (left panel) and ^mCherry^ZFP36L1^+^(right panel) CD8 T cells 6 h after stimulation with peptides at indicated concentrations. (B) Average expression per cell shown as geo mean of the expression of ^mAmetrine^ZFP36 (left panel) and ^mCherry^ZFP36L1 (right panel) when gated on the positive population. (C) Representative histograms show expression ^mAmetrine^ZFP36 (left panel) and ^mCherry^ZFP36L1 (right panel), when stimulated with 10 nM N4, V4, or T4 peptide. (D) Relative expression (RQ) of ^mAmetrine^ZFP36 and ^mCherry^ZFP36L1. RQ was calculated as average mean expression of the fluorescent proteins per cell (geo mean) divided by their average expression when cells were stimulated with 10 nM N4 peptide, which represents their maximal expression. Data in (A) and (B) are compiled from three biological replicates and are representative of three independent experiments, two of which included stimulation with Q4 peptide. Error bars indicate the standard deviation from the mean. Data in (D) are compiled from three independent experiments. Shaded area indicates the standard deviation from the mean. (E) Frequencies of co‐transferred OT‐I cell in spleens of recipient mice, 7 days after infection with *attLm*‐OVA. Data are compiled from two independent experiments. Statistical significance was determined by two‐way ANOVA followed by Tukey's test for multiple comparisons. (F) Representative histograms of pSTAT5 and (G) CD25 in WT cells co‐cultured with GFPZFP36L1 (green histogram) or ZFP36L1 KO (blue histogram) cells 48 h after activation with plate‐bound CD3 and CD28. h) Quantification of pSTAT5 i) CD25 expression. Each data point represents a technical replicate, and data are representative of two independent experiments. Statistical significance was tested by paired *t*‐test. All error bars indicate the standard deviation from the mean. The statistical mean is indicated by the horizontal line in all panels.

We stimulated naïve CD8 T cells from OT‐I ^mAmetrine/+^ *Zfp36* ^mCherry/+^ *Zfp36l1* heterozygous mice with variants of SIINFEKL peptide, which have altered stimulation potency of the OT‐I TCR but bind equally to H‐2K^b^. For stimulation, we used splenocytes pulsed with different concentrations of high‐affinity N4 peptide and its low‐affinity variants Q4 (SIIQFEKL), T4 (SIITFEKL), which marks the threshold of positive and negative selection, and V4 (SIIVFEKL) which has a 1000 times lower potency compared with N4 [49, 50]. We measured the expression of ^mAmetrine^ZFP36 and ^mCherry^ZFP36L1 by flow cytometry. Frequencies of activated cells positive for ^mAmetrine^ZFP36 reached maximal levels with 0.3 nM of N4, Q4, and T4, but required 3 nM V4 (Fig. 6A). In contrast, higher amounts of the lowest affinity peptide, (10 nM V4), failed to induce a maximal frequency of ^mCherry^ZFP36L1 expressing cells (Fig. 6A). Strikingly, the different behavior in expression was especially evident when the mean fluorescence intensity per cell of each marker was analyzed. High amounts of N4, Q4, and T4 peptides induced maximal expression of ^mAmetrine^ZFP36 per cell, as the fluorescence intensity reached a plateau irrespective of peptide affinity (Fig. 6B). By contrast, ^mCherry^ZFP36L1 expression never reached the maximal levels observed after N4 stimulation even at high concentrations of Q4, T4, and V4 (Fig. 6B). ^mCherry^ZFP36L1 expression per cell after stimulation with 10 nM peptide was linearly related to peptide affinity and showed discriminative sensitivity for peptide variants (Fig. 6C, D). This prompted us to test if both ZFP36 and ZFP36l1 are required for T‐cell competitiveness in response to high‐affinity antigens during infection. Therefore, we cotransferred naive OT‐I specific WT cells with OT‐I cells that lacked either ZFP36 or ZFP36L1 and infected the recipient mice with *attLm*‐OVA. Although CD45.1 WT and CD45.2 WT or ZFP36 KO OT‐I cells expanded to the same extent by day 7 in the spleen of recipient mice, cells lacking only ZFP36L1 recapitulated the phenotype of the dKO cells in competition with WT OT‐I cells (Fig. 6E).

To test further the role of ZFP36L1 in promoting STAT5 signaling we co‐cultured naïve WT CD8 T cells with naïve CD8 T cells from transgenic mice which express a GFPZFP36L1 fusion protein (*GFPZfp36l1*) [51]. This model of forced expression of ZFP36L1, which is independent of TCR signaling, in addition to the endogenous expression of ZFP36L1 in these T cells, can be regarded as an overexpression system which we predicted to show the opposite effects on STAT5 signaling to the knockout. After activation with plate‐bound anti‐CD3 and anti‐CD28 for 48 h we found pSTAT5 as well as CD25 expression in GFPZFP36L1 cells was greater than that in WT cells (Fig. 6F–I). The opposite result was observed in ZFP36L1 KO cells where the expression of pSTAT5 and CD25 was reduced when compared with WT (Fig. 6F–I). Taken together, these data show that ZFP36L1 promotes signaling via STAT5.

### *Socs1* limits the competitiveness of low‐affinity T cells

**Figure 7:**
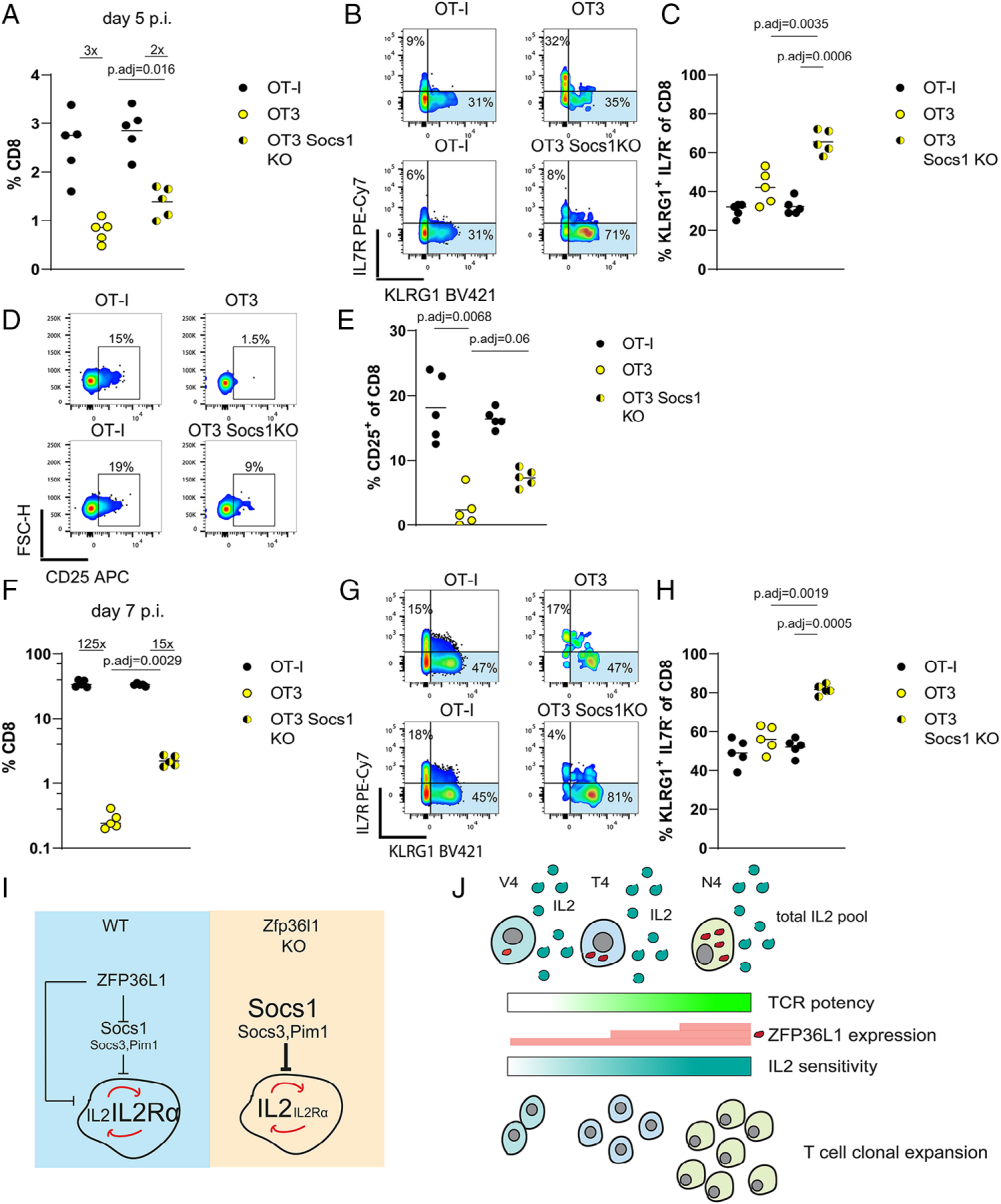
*Socs1* limits the competitiveness of low‐affinity T cells. (A) Frequencies of total CD8 cells from co‐transferred OT‐I and OT‐3 cells or OT‐I and *Socs1* deficient OT‐3 cells, in blood of recipient mice on day 5 after infection with *attLm*‐OVA. (B) Representative FACS plots and frequencies (C) of KLRG1^+^ cells in blood of mice 5 days after infection. (D) Representative FACS plots and frequencies (E) of CD25 positive cells. (F) Frequencies of CD8 cells from co‐transferred OT‐I and OT‐3 cells which have been treated with nontargeting guide RNAs or OT‐I and *Socs1* deficient OT‐3 cells, in blood of recipient mice on day 7 after infection with *attLm*‐OVA. (G) Representative FACS plots and frequencies (H) of KLRG1^+^ cells in blood of mice 7 days after infection. Fold differences between frequencies of cells are indicated in panels (A) and (F). The statistical mean is indicated by a horizontal line in all panels. Data is representative of two independent experiments. Statistical significance was determined by two‐way ANOVA followed by Tukey's test for multiple comparisons. Fold changes of the difference are indicated. (I) Schematic model of ZFP36L1 comprising a crucial part of the IL‐2 signaling pathway as part of an incoherent feed‐forward regulatory loop. (J) Schematic illustration of ZFP36L1 governing the selection and outgrowth of high‐affinity clones based on their sensitivity to IL‐2.

We next addressed whether enhancing the response to cytokines during infection would improve the competitive fitness of low‐affinity CD8 T cells. We used OT‐3 CD8 T cells which express a transgenic TCR with a 100‐fold lower affinity for SIINFEKL than OT‐I [52]. Stimulation of naive OT‐3 with N4 peptide resulted in a similar amount of ZFP36L1 as in naïve OT‐I cells stimulated with the low‐affinity peptide T4 (Fig. S5A, B). Co‐transfer of OT‐I and OT‐3 cells followed by infection with *attLm*‐OVA resulted in a 3‐fold difference of transferred cell frequencies in the blood of recipient mice on day five postinfection (Fig. 7A). This difference was only slightly less upon co‐transfer OT‐I cells with OT‐3 cells with deleted *Socs1* (Fig. 7A). The deletion of *Socs1* in OT‐3 cells led to an increased accumulation of KLRG1^+^ SLEC on day 5 postinfection (Fig. 7B, C) and an increased expression of CD25 on the surface of these cells (Fig. 7D, E). At the peak of the primary response, the disadvantage of OT‐3 was more than a 100‐fold difference (Fig. 7D). However, when OT‐3 cells lacked *Socs1* this difference was significantly reduced (15‐fold) (Fig. 7F), suggesting that deletion of *Socs1* increases competitive fitness of low‐affinity CD8 T cells during the later expansion phase of the primary response. Thus, this result resembled the results observed with *Socs1* deletion in dKO OT‐I cells. On day 7, low affinity T cells lacking *Socs1* showed a greater tendency to form SLEC which is in contrast to WT OT3 cells which start to contract and show reduced frequencies of KLRG1^+^ cells compared with WT OT‐I (Fig. 7G, H). This suggests that *Socs*1 limits expansion and differentiation into SLEC, which can be attributed to the attenuation of cytokine signaling. In summary, we suggest that lifting the limits of cytokine signaling also enhances the competitiveness of low‐affinity CD8 T cells, and therefore differential expression of ZFP36L1 should also contribute to their selection.

## Discussion

This work establishes ZFP36L1 as a mechanistic link between TCR affinity and the responsiveness to IL‐2. Moreover, this work explains previously observed priming defects of T cells and their failure to be efficiently recruited into immune responses in the absence of multiple ZFP36 family members [29, 30]. Here, we show a T‐cell intrinsic mechanism that reveals ZFP36L1 to be a nonredundant regulator of cytokine signaling in T cells and a key driver of cytokine‐driven clonal expansion.

The ability of the RBP to establish the competitive advantage of high‐affinity T cells is most apparent when RBP‐deficient high‐affinity OT‐I cells have to compete with an equal number of high‐affinity WT OT‐I T cells. The competitive disadvantage of cells lacking RBPs is much less pronounced when high‐affinity dKO OT‐I T cells have to compete with endogenous T cells, with varying affinities for OVA‐derived antigens. The number of endogenous SIINFEKL K^b^‐specific T cells is estimated to be no more than a few hundred [53] and is composed of a range of affinities for SIINFEKL peptide. In line with this, we find it striking that the expression of ZFP36L1 is highly responsive to the affinity of TCR peptide MHC‐I interaction. We observed that antigen dose did not saturate the expression of ZFP36L1 per cell. The high sensitivity of ZFP36L1 expression to changes in affinity suggests that it can act as a rheostat guiding the selection of high‐affinity clones by promoting their responsiveness to IL‐2. Similar TCR‐responsive expression behavior has been reported for *Irf4* which converts TCR signals in an analog fashion and drives metabolic reprogramming and expansion of CD8 T cells [12, 54]. This contrasts with Myc which shows invariable expression per cell in response to TCR stimulation strength [11] and Nur77 which marks an invariable activation threshold for T cells [10].

ZFP36 and ZFP36L1 limit the expression of cytokines including IL‐2 [30], whereas ZFP36L1 specifically acts on the JAK‐STAT5 pathway downstream of common γ‐chain cytokine receptors. In this way ZFP36L1 is a crucial part of a type 2‐incoherent feed‐forward loop [55] controlling the IL‐2 signaling axis (Fig. 7I). In WT cells the IL‐2 regulatory circuit would critically depend on the exogenous input of IL‐2 to shift the equilibrium to enhance IL‐2 signaling. ZFP36L1 suppresses IL‐2 production and at the same time increasesg sensitivity to it by suppressing a set of inhibitors of cytokine signaling. In the absence of ZFP36L1 sensitivity to IL‐2 is lost progressively, ultimately resulting in premature downregulation of CD25 and termination of a potent IL‐2 signal. This would be manifested most strikingly when IL‐2 becomes limiting during the course of an immune response [46] which results in reduced clonal expansion and is observed in the later expansion phase. ZFP36L1 could act in concert with the transcription factor IRF4 which shows similar TCR‐dependent regulation and promotes the cytokine response by inducing the expression of the β‐chain of the IL‐2 receptor CD122 [56].

It has previously been shown that the affinity of the TCR does not necessarily drive the rate of initial cell division but that high‐affinity T cells proliferate and expand for an extended period of time [15]. Extended expansion of T cells is driven by cytokines including IL‐2. This becomes rate‐limiting during the course of an infection and efficient competition for IL‐2 requires the persistent expression of CD25 [16] which can be enhanced by other cytokines such as type‐I IFNs and IL‐12 [8] or suppressed by cytokines such as IFNγ [33] and promotes additional rounds of cell division. The ability of inflammatory cytokines to rescue high but not low‐affinity clones from apoptosis has been suggested as a mechanism for preferential outgrowth of high‐affinity clones when antigen availability is limited [17]. We show that ablation of *Socs1* not only restored the competitiveness of dKO cells but also that of T cells bearing a low‐affinity TCR in competition with high‐affinity clones. This highlights that efficient sensing of IL‐2, and potentially other inflammatory signals, including IL‐12 and IFNs, is principally acting during competition and selection of high‐ and low‐affinity T cells.

Cytokines drive cell expansion but additionally regulate differentiation. IL‐2 has a critical role in the formation of SLEC, and prolonged expression of CD25 is associated with greater progression toward terminal differentiation [16, 46]. In our current and previous work [30], we find that RBP‐deficient CD8 T cells more rapidly form SLEC. This happens despite a reduced response to IL‐2 which we describe in the present study to be mainly responsible for the reduced expansion of antigen‐specific cells. We find that the deletion of *Socs1*, which enhances IL‐2 signaling and restores the ability of cells to expand further accelerates SLEC formation in RBP‐deficient CD8 T cells. We have previously reported that the *Zfp36/Zfp36l1*‐deficient CD8 T cells are less dependent on costimulation and acquire effector functions earlier. We suggested a mechanism whereby these RBPs limit expression of differentiation‐promoting transcription factors including Irf8, *Notch1*, and NF‐kB family members. We suggest that the effector differentiation pathways suppressed by the RBPs are not exclusively driven by IL‐2 [30]. Strikingly, full acquisition of effector function has been shown to take place faster in low‐affinity T cells, although these cells have a shorter exposure to antigens and cytokines such as IL‐2 [57]. Thus, effector differentiation and expansion of low‐ and high‐affinity T cells can be uncoupled.

The limiting of autocrine IL‐2 production and the simultaneous de‐repression of IL‐2 signaling suggests that ZFP36L1 enforces dependence of CD8 T cells on paracrine IL‐2, which becomes available during the early phase of an infection and is largely produced by antigen‐specific CD4 T cells and DCs [58]. We suggest that expression of ZFP36L1 in activated T cells significantly decreases autonomy during priming, increasing the dependence on costimulation but rendering them competitive with CD4 effectors or regulatory T cells for available IL‐2 pools. In a situation where stimulation of T cells of varying affinities by antigen is sufficient to induce activation and proliferation, lesser expression of ZFP36L1 will position lower‐affinity T cells at a disadvantage to compete for cytokines, whereas at the same time safeguard the costimulatory dependency and thus control/deletion of autoreactive and harmful clones (Fig. 7J). Even small differences in IL‐2 sensitivity would result in the progressive loss of inferior clones; these would be outcompeted by more sensitive clones which are then further rewarded by having expanded more thus establishing a competitive exclusion principle.

## Materials and methods

### Mice

Mice with single or combined floxed *Zfp36* and *Zfp36l1* alleles [59, 60], B6.Cg‐Tg(CD4‐cre)1Cwi [61] mice, GFP*Zfp36l1* [51] and OT‐I [62] TCR (Vα2 and Vβ5 recognizing peptide residues 257–264 of chicken ovalbumin in the context of H2K^b^) were generated on the C57BL/6 background at the Babraham Institute. The B6.SJL‐*Ptprc^a^Pepc^b^*/BoyJ (CD45.1) mice were bred at the Babraham Institute. OT3 TCR‐Vβ5 transgenic B6.SJL‐*Ptprc^a^Pepc^b^*/BoyJ (CD45.1) mice on a TCRα KO background carrying a TCR‐Vα transgene expressing IRES‐GFP [52] were a kind gift by Dietmar Zehn and were bred at the Babraham Institute. CD45.1 CD45.2 double‐positive mice were bred as F1 from C57BL/6 (CD45.2) and B6.SJL‐*Ptprc^a^Pepc^b^*/BoyJ (CD45.1) mice at the Babraham Institute. ^mAmetrine^ *Zfp36* and ^mCherry^ *Zfp36l1* mice were generated by Cyagen bioscience on a C57BL/6 background and maintained in the Babraham Institute Biological Support Unit.

Targeting vectors were designed to harbor the sequence encoding mAmetrine or mCherry without stop codon, in‐frame upstream of the ATG translation start site of *Zfp36* or *Zfp36l1*, respectively. The *Zfp36l1* targeting vector featured diphtheria toxin A, a neomycin resistance cassette flanked by *Frt* recombination sequences and homology arms which were amplified from a mouse genomic BAC clone. The ZFP36 targeting vector contained self‐deletion anchor sites instead of *Frt* recombination sites. When homozygous, we did not observe lethality or any pathology in the reporter mice, suggesting a normal function of the proteins, deficiency of which causes an inflammatory syndrome [63] or embryonic lethality [64] in mice.

No primary pathogens or additional agents listed in the FELASA recommendations have been confirmed during health monitoring since 2009. The ambient temperature was ∼19–21°C and the relative humidity was 52%. Lighting was provided on a 12 h light: 12 h dark cycle including 15 min “dawn” and “dusk” periods of subdued lighting. After weaning, mice were transferred to individually ventilated cages with 1–5 mice per cage. Mice were fed CRM (P) VP diet (Special Diet Services) ad libitum and received seeds (e.g. sunflower, millet) at the time of cage‐cleaning as part of their environmental enrichment. All mouse experimentation was approved by the Babraham Institute Animal Welfare and Ethical Review Body. Animal husbandry and experimentation complied with existing European Union and United Kingdom Home Office legislation.

In our in vitro experiments, naïve CD8 T cells were isolated from male and female mice. For in vivo experiments we used female mice as a source for the naïve CD8 donor T cells. As recipient mice in cell‐transfer experiments, both male and female mice were used.

### Infection with *Listeria monocytogenes*

Eight‐to‐fourteen‐week‐old male and female mice were used for all experiments. Bacteria were grown in a BHI medium to an OD_600_ of 0.1 before each experiment. Mice were infected with a sublethal dose of 5 × 10^6^ CFU attenuated (ΔactA) *L. monocytogenes* expressing OVA_(257–264)_ [30] by intravenous administration.

Adoptive transfer experiments: naive OT‐I cells (if not otherwise stated) were sorted by Flow Cytometry from spleens and LN of mice from a respective genotype and a total of 1000 cells per mouse was transferred intravenously on the day before infection. In all co‐transfer experiments, donor cells were mixed with 1 × 10^6^ carrier splenocytes of the same genotype as the host.

For characterization of transferred OT‐I cells, after transfer mice were bled on indicated dates and spleens were collected at final points of analysis. For analysis of cytokine production, cells were incubated with 10^−7^M N4 peptide for 3 h in the presence of Brefeldin A (1 μg/mL) in full cell culture medium (IMDM including 10%FCS 50 μM beta β‐Me). After surface staining, cells were fixed with 2% PFA for 20 min at 4°C and permeabilized with BD Perm/wash +1% ‐FCS for 20 min at 4°C, before intracellular cytokine staining.

### In vitro culture and activation of T cells

All in vitro and cell culture experiments were performed in IMDM culture medium (Gibco) supplemented with 10% FCS, GlutaMAX (Gibco), 40U penicillin/streptomycin (Gibco), and 50 μM β‐mercaptoethanol.

Naive CD8 T cells were isolated from spleen and LN using two rounds of negative depletion with Streptavidin DynaBeads (Thermo Fisher) using 1 × 10^8^ and 4 × 10^7^ beads per 1 × 10^8^ total splenocytes/LN cells. The following antibodies were used for the depletion cocktail: B220‐Bio (RA3‐6B3), CD4‐Bio (GK1.5), CD11b‐Bio (M1/70), CD11c‐Bio (N418), CD19‐Bio (1D3), CD44‐Bio (IM7), CD105‐Bio, F4/80‐Bio (BM8), GR1‐Bio (RB6‐8C5), NK1.1‐Bio (PK136), Ter119‐Bio (Ter‐119), and γδTCR‐Bio (GL3).

Cells were stimulated at a starting concentration of 5 × 10^5^ cells/mL, if not otherwise stated with 5 μg/mL plate‐bound anti‐CD3 (145‐2C11, BioXcell) and 1 μg/mL plate‐bound anti‐CD28 (37.51, BioXcell). OT‐I transgenic naive CD8 T cells were stimulated with (N4) SIINFEKL peptide at 10^−10^ M if not otherwise stated. The altered peptide ligands SIIQFEKL, SIITFEKL, and SIIVFEKL were used to stimulate OT‐I cells in some experiments. In some conditions, recombinant mouse IL‐2 and IL‐12 (Peprotech) were added at 20 ng/mL and 2.5 ng/mL, respectively, or at concentrations as indicated. Anti‐mouse IL‐2 (JES6‐1A12, BioXcell) was used at 50 μg/mL to block autocrine IL‐2. Anti‐mouse‐IFNγ (XMG1.2, BioLegend) and anti‐mouse‐TNF (MP6‐XT22, BioLegend) were used at 10 and 25 μg/mL, respectively. Recombinant human IL‐2 (Peprotech) was added at indicated concentrations.

For the analysis of proliferation, cells were labeled with cell trace violet (Thermo Fisher) or cell trace yellow (Thermo Fisher) at 10 μM final concentration for 6 min at 37°C, before stimulation. Cell numbers per generation were enumerated using cell counting beads for flow cytometry (ACBP‐50‐10, Spherotech).

### CrispR Cas9 RNP‐mediated gene editing

For electroporation of Cas9 RNP, naïve OT‐I T cells were isolated using the StemCell EasySep Mouse Naïve CD8^+^ T‐cell isolation kit (StemCell; 19853). Isolated cells were electroporated with complexes of Cas9 and control or IL‐2 targeting gRNA (all from Integrated DNA Technologies) in OptiMEM medium (Gibco) using the NEPA21 electroporator (Nepagene). After electroporation cells were cultured for 24 h in RPMI medium (Gibco) containing 10% FCS and 10 ng/mL recombinant IL‐7 (Peprotech; 217‐17), before transfer into recipient mice. The following guide sequence (Integrated DNA Technologies) was used to target the indicated genomic sequence of the *Socs1* gene: AAGTGCACGCGGATGCTCGT, *Il2* gene: AAGATGAACTTGGACCTCTG *Cish* gene: CTTGTCAAGACCTCGAATCC. Gene editing efficiency was assessed in vitro. Cas9 targeted cells were maintained in culture for 48 h in the presence of 10 ng/mL IL‐7 and restimulated with 10^−7^ M N4 peptide in the presence of 20 ng/mL IL‐2. Cells were expanded for 6 days in culture in the presence of IL‐2. Protein expression of targeted genes was assessed by western blotting.

### Flow cytometry and antibodies

For cell surface staining single cell suspensions from tissues or cultured cells were prepared in FACS buffer containing 1× PBS, 1% FCS ±2 mM EDTA (if not otherwise stated in the methods). All cells were blocked with Fcγ blocking antibody (24G2, BioXcell) and incubated with fixable cell viability dye eF780 (ThermoFisher or BD) for 20 min at 4°C. For intracellular staining, cells were fixed with BD Cytofix/Cytoperm (554722) or 2%–4% PFA for 20 min at 4°C. Cells were permeabilized with BD Permwash (554723) containing 1% FCS for 20 min at 4°C. Intracellular staining was performed in BD Permwash containing 0.5% FCS and the intracellular antibody cocktail for 1 h at RT. Surface‐stained cells from infection experiments were fixed with BD Cytofix/Cytoperm for 30 min at 4°C before analysis. Staining for pSTAT5 and antiapoptotic molecules was performed by fixing cells immediately during stimulation with 4% PFA at final concentration of 2% on ice. Cells were fixed for 30 min. Cells were permeabilized with 90% methanol for 30 min on ice and stored at −20°C. Cells were finally stained in 1× PBS + 0.05% BSA for 1 h on ice.

The following antibody clones were used in the flow cytometry experiments: CD8 (53‐6.7;1:400), CD45.1 (A20; 1:100), CD45.2 (104;1:100), KLRG1 (2F1;1:400), CD127 (A7R34;1:100), CD44 (IM7;1:400), CD25 (7D4, PC61;1:400), CD69 (H1.2F3;1:400), TCRβ (H57‐597;1:200), Ki‐67(SolA15), Mcl1 (LUVBKM; 1:20), Bcl2 (BCL/10C4; 1:50), pSTAT5 (47;1:5)), activeCaspase3 (C92‐605;1:100), and ZFP36L1 (E6L6S;1:1000). CD16/32 2.4G2 BioXcell BE0008 (1:2000, 0.5 ug/mL). Donkey anti‐Rabbit AF647 (711‐605‐152; 1:1000). Data were acquired using a Fortessa flow cytometer equipped with 355, 405, 488, 561, and 640 nm lasers (Beckton Dickinson). Flow cytometry data were analyzed using FlowJo 10.6 software.

### Western blotting

Whole‐cell lysates were prepared by resuspending CTL cell pellets in 2× Laemmli buffer containing 5% β2‐mercaptoethanol. The equivalent of 1–2 million cells were loaded per lane. Protein concentrations were determined by BCA protein assay (Pierce, 23225). Samples were resolved by 12% SDS‐PAGE and transferred to nitrocellulose membrane using an iBlot2 transfer device (IB21001). The following antibodies were used in western blotting experiments: Rabbit anti‐CISH (D4D9; 1:100; Cell Signalling), Goat polyclonal anti‐SOCS1 (ab9870; 1:500; Abcam), mouse anti‐Tubulin (DM1A; 1:10,000; Sigma). Secondary HRP‐conjugated antibodies: anti‐goat HRP Trueblot (1:2000) (ebioscience), anti‐Rabbit HRP (1:10,000; DAKO), mouse anti‐ZFP36 (Origene #OTI3D10, 2 μg/mL), Rabbit‐anti ZFP36L1 (CST #BRF1/2, 32 ng/mL), Rabbit anti‐GAPDH (CST #5174, D16H11, 1:1000). Secondary antibodies for detection with Licor used: anti‐mouse IgG IRDye800CM (Licor #926‐32210) and anti‐Rabbit IgG IRDye680RD (Licor #925‐68071). Membranes were scanned using Licor Odyssey CLx using standard methods, or ECL prime (Cytiva, RPN2232) for HRP‐conjugated antibody detection. Image analysis was conducted using ImageStudio Lite version 5.2, and normalized protein signal was calculated using standard methods.

### RNA sequencing

500.000 OT‐I‐transgenic naïve CD8+ T cells were stimulated in 200 μL IMDM medium containing 10% FCS, 50 μM beta‐Me, and 40U penicillin/streptomycin with (N4) SIINFEKL peptide at 10^−10^ M, for 0–16 h. Total RNA was extracted using the RNeasy Micro Kit (Qiagen, REF:74004). Opposing strand‐specific RNA‐seq libraries were generated using the SMARTer Stranded Total RNA‐Seq Kit v3 ‐ Pico Input Mammalian (Takara), according to the manufacturer's instructions, and sequenced on an Illumina NovaSeq 6000 using 150bp paired‐end reads.

### Data and statistical analysis

Statistical analysis was performed using Graph Pad Prism 9.3.1 and Microsoft Excel. RNA‐seq data from in vitro‐activated CD8^+^ T cells was trimmed using Trim Galore (https://www.bioinformatics.babraham.ac.uk/projects/trim_galore), first to remove poor quality and adapter sequence from the 3′ end of all reads and second to remove 14 bases from the 5′ end of read 2 and 11 bases from the 3′ end of read 1, to ensure removal of the in‐line UMI sequence comprising the start of read 2. Reads were mapped to the GRCm39 mouse genome using HISAT2 (v2.1.0), suppressing unpaired or discordant alignments and considering known splice sites from the Ensembl GRCm39 v103 annotation. Note that this resulted in a much lower mapping efficiency and thus fewer mapped reads than expected; coupled with our quality control results and further mapping to rRNA sequences using Bowtie, substantial ribosomal RNA contamination was identified as the cause of this low mapping efficiency, suggesting inefficient depletion during library preparation. The quality and consistency between replicates for the successfully mapped reads, however, appeared good, therefore we continued with the analysis. Raw read counts over mRNA features were quantified using Seqmonk (https://www.bioinformatics.babraham.ac.uk/projects/seqmonk). Differentially expressed genes at the different time points after CD8^+^ T‐cell activation were identified using DESeq2 analysis with default parameters, comparing dKO with WT for each time point, and significantly increased genes were selected for adjusted *p*‐value ≤ 0.05 and log2 FC > 0.3 (using “normal” log2 fold change shrinkage). ZFP36/L1 library of CLIP targets for T cells was collated as previously described [30]. CLIP data were visualized using the following application: https://github.com/LouiseMatheson/iCLIP_visualisation_shiny.

## Conflict of interest

Some research in M.T.’s lab is funded by Astra Zeneca. The remaining authors declare no financial or commercial conflict of interest.

## Author contributions

Georg Petkau: conceptualization, methodology, investigation, validation, formal analysis, visualization, and writing the original draft. Twm J. Mitchell, Marian Jones Evans, and Fiamma Salerno: methodology, validation, review, and editing. Louise Matheson: software, formal analysis, investigation, visualization, review, and editing. Martin Turner: conceptualization, supervision, funding acquisition, writing, review, and editing.

### Peer review

The peer review history for this article is available at https://publons.com/publon/10.1002/eji.202350700

**Abbreviations**
CLIP: crosslink‐immunoprecipitation
RBPs: RNA‐binding proteins
SLEC: short‐lived effector cells

## Supporting information

Supporting Information

Supporting Information

## Data Availability

Sequencing data of time course gene expression in WT and dKO CD8 T cells is publicly available on: GEO: GSE230373.
