# Supplementary material for: Zfp36l1 establishes the high‐affinity CD8 T‐cell response by directly linking TCR affinity to cytokine sensing
Source: Eur J Immunol. 2023 Dec 7;54(2):2350700. doi: 10.1002/eji.202350700 (PMC11146077; doi:10.1002/eji.202350700)
Supplement: Supplementary file 1 — Supporting Information [file EJI-54-2350700-s002.pdf]

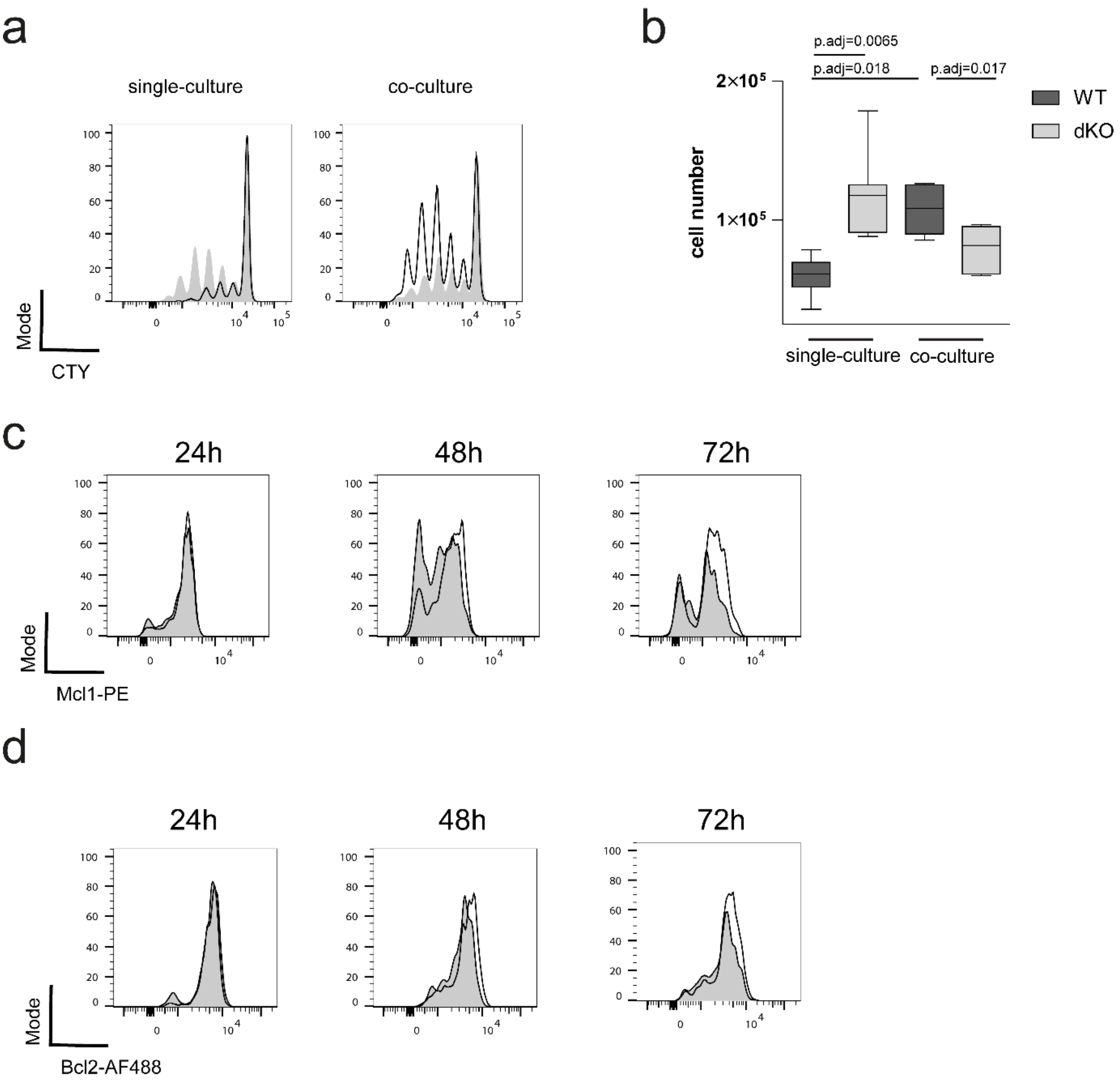

Supplementary Figure 1: RBP deficient T cells promote the WT T cell response in co-culture. **a)** Representative panels show the dilution of CTY by naïve WT (open) and dKO (filled) CD8 T cells stimulated with plate bound anti CD3 and anti CD28 for 72h individually or in co-culture **b)** Absolute cell numbers of WT and dKO cells after 72 hours with plate bound anti CD3 and anti CD28 in individual and co-culture. Data is compiled from 7 independent experiments. **c)** Representative histograms show Mcl1 and d) Bcl2 expression in WT (open histograms) and dKO (filled histograms) in co-cultures activated for indicated times with peptide. Statistical significance was determined by one-way ANOVA followed by Tukey`s test for multiple comparisons. Box Plots in **b)** indicate the data distribution by showing the min and max values around the statistical mean.

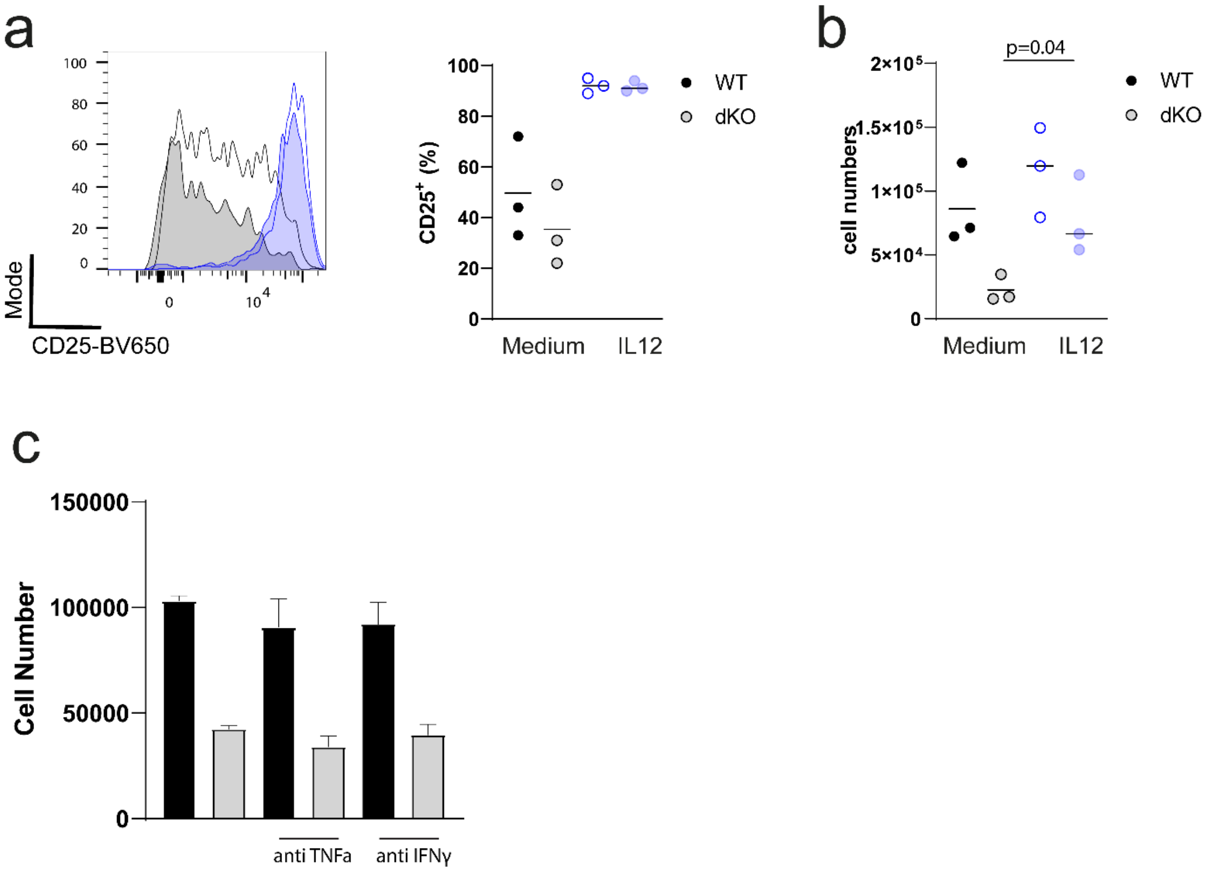

Supplementary Figure 2: IL12 partially restores dKO T cell competitiveness.

a) Representative histograms show the expression of CD25 by WT (open) and dKO (filled) CD8 T cells stimulated with N4 in the presence (blue) or absence of IL-12. **b)** Absolute cell numbers of WT and dKO OT-I cells after 72 hours stimulation with N4 peptide in co-culture in the presence or absence of IL-12. **c)** Cell numbers of WT and dKO OT-cells cultured for 72h in the presence of anti TNF or anti IFNγ antibodies. Error bars indicate the standard deviation of the mean of n=3 technical replicates. Statistical significance was determined by unpaired t-test. The statistical mean is indicated by horizontal line in all panels.

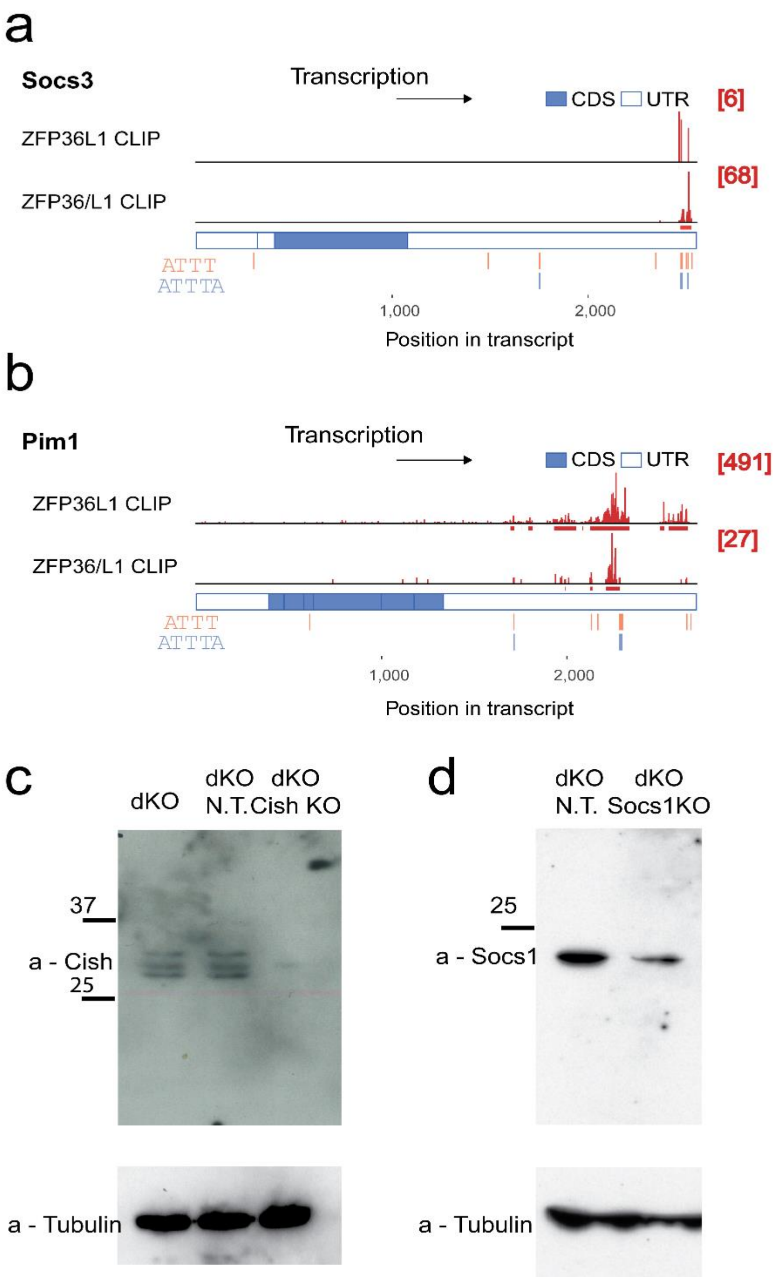

Supplementary Figure 3: Socs3 and Pim1 are direct RBP CLIP targets and validation of Cas9 deletion of *Cish* and *Socs1*

a) CLIP data showing number and position of sequencing reads (in red) across *Socs3* and *Pim1* **b)** transcripts (a set of top two lanes). In each set top lane shows ZFP36L1 CLIP data from OT-I CD8 CTLs stimulated for 3h with N4 peptide; bottom lane shows panZFP36 family CLIP data from in vitro activated naive CD4 T cells. ATTT and ATTTA motifs are identified in orange and blue. **c)** Validation of deletion of *Cish* and **d)** *Socs1* by western blot in OT-I CTLs differentiated for 6 days in the presence of IL-2 after stimulation with N4 peptide. Lanes were loaded with lysates from dKO cells treated with non-targeting guides or specific guides for *Cish* and *Socs1* as indicated.

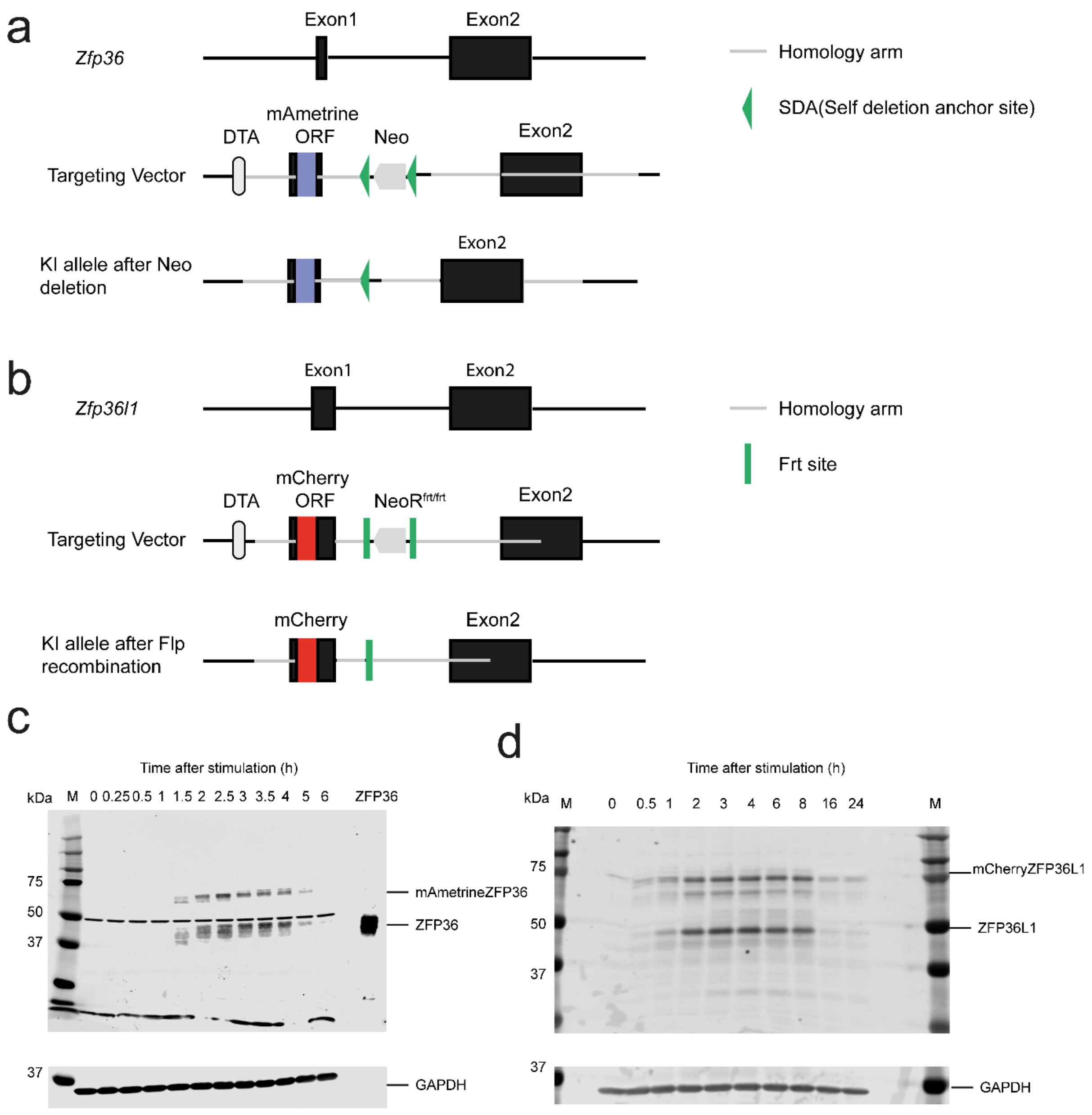

Supplementary Figure 4: ZFP36 and ZFP36L1 fluorescent reporter mice. a) Schematic illustrates the targeting vector and the resulting engineered ^{mAmetrine}ZFP36 allele and ^{mCherry}ZFP36L1 **b)** allele where the respective gene sequences encoding the fluorescent reporter proteins have been knocked into the first exons of *Zfp36* and *Zfp36l1*. Peptide sequences with highlighted fluorescent reporters are indicated for each fusion protein. Detection of endogenous and fusion protein expression of ZFP36 and ZFP36L1 from heterozygous mice. **c)** Western blot of ZFP36 and ^{mAmetrine}ZFP36 and **d)** ZFP36L1 and ^{mCherry}ZFP36L1 expression in *in vitro* expanded CD8 T cells from heterozygous mice stimulated with 10^{-10}M N4 peptide for the indicated times. Equal amounts of protein were loaded per lane. Last lane in **c)** was loaded with lysate from HEK293 cells expressing ZFP36. Each blot is a representative of at least three independent experiments.

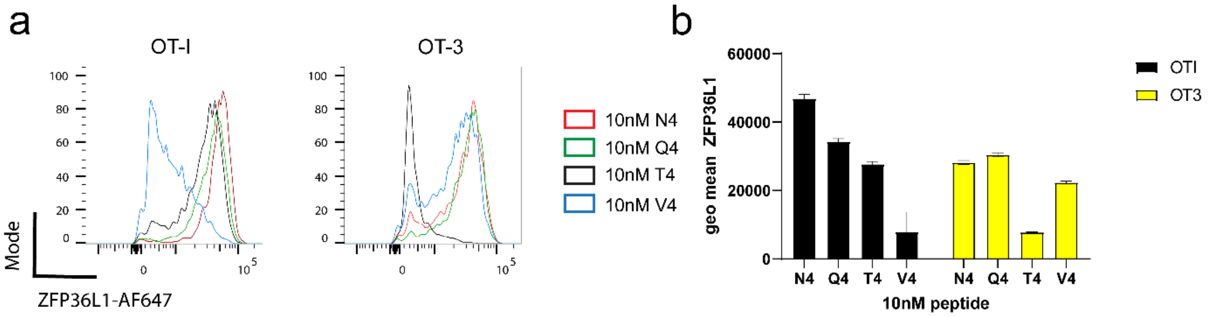

Supplementary Figure 5: ZFP36L1 expression in OT-3 cells. a) Representative histograms show expression of ZFP36L1 detected by intracellular antibody staining in naïve OT-I and OT-3 cells stimulated with 10nM of different OVA peptides for 4 hours. **b)** Average expression per cell shown as geo mean of the expression of ZFP36L1, when gated on the positive population. All error bars indicate the standard deviation from the mean.
